# Proponent or collaborative: Physician perspectives and approaches to disease modifying therapies in sickle cell disease

**DOI:** 10.1371/journal.pone.0178413

**Published:** 2017-07-20

**Authors:** Nitya Bakshi, Cynthia B. Sinha, Diana Ross, Kirshma Khemani, George Loewenstein, Lakshmanan Krishnamurti

**Affiliations:** 1 Division of Pediatric Hematology-Oncology, Children’s Hospital of Pittsburgh, Pittsburgh, Pennsylvania, United States of America; 2 University of Pittsburgh, Pittsburgh, Pennsylvania, United States of America; 3 Aflac Cancer and Blood Disorders, Children’s Healthcare of Atlanta, Atlanta, Georgia, United States of America; 4 Division of Pediatric Hematology-Oncology-BMT, Department of Pediatrics, Emory University, Atlanta, Georgia, United States of America; 5 Department of Social and Decision Sciences, Carnegie Mellon University, Pittsburgh, Pennsylvania, United States of America; University of Liverpool, UNITED KINGDOM

## Abstract

Sickle cell disease (SCD) is an inherited blood disorder that primarily affects African-American and other ethnic minority populations. There are three available disease-modifying therapies for sickle cell disease: hydroxyurea (HU), bone marrow transplantation (BMT), and chronic blood transfusion (CBT). Since these treatments vary in their therapeutic intent, efficacy in preventing progression of the disease, short and long-term adverse effects, costs and patient burden, the decision-making process regarding these therapies is complicated for both the patient and healthcare provider. While previous research has focused on the patient perspective of treatment-related decision making, there is a paucity of research investigating the physician perspective of treatment-related decision making. We conducted a qualitative study with physicians who were experts in the field of SCD. Interviews focused on physician perceptions of patient decisional needs as well as physicians’ approach to decision making regarding disease-modifying therapies in SCD. Thirty-six physician interviews were analyzed, with a focus on their perspectives regarding available treatment options and on how they approach decision making with patients. We identified two narrative approaches. The Collaborative approach (CA) was characterized by emphasizing the need to discuss all possible treatment options to ensure that the patient and/or family was equipped to make an informed decision. The Proponent approach (PA) was characterized by strongly advocating a pre-determined treatment plan and providing patients/families with information, with the objective of convincing them to accept the treatment. An interplay of patient-related and disease-related factors, decision type and physician-related factors, as well as institutional frameworks, influenced physician perspectives on treatment options and decision making regarding these therapies. These findings point to the potential value of developing systems to foster patient engagement as a way of facilitating shared decision making.

## 1. Introduction

Approximately 100,000 individuals in the U.S. live with sickle cell disease (SCD). SCD is an inherited disorder that causes red blood cells to take on a sickle-like shape. These abnormally shaped blood cells can impair circulation, resulting in vaso-occlusive events that are the hallmark of the disease. Clinically, SCD is characterized by anemia and episodes of severe vaso-occlusive pain, and it can cause complications such as strokes, acute chest syndrome, splenic sequestration, and increased risk of bacterial sepsis[1]. Furthermore, SCD can cause long-term complications secondary to end-organ damage, particularly to the central nervous system, eye, lungs, bones, and kidneys. Overall, SCD is associated with significant impairment of quality-of-life[2] and premature mortality[3]. Notably, the vast majority of individuals with SCD in the U.S. are African-American and other ethnic minority populations.

There are three available disease-modifying therapies for sickle cell disease: hydroxyurea (HU), bone marrow transplantation (BMT), and chronic blood transfusion (CBT). These treatments vary in their therapeutic intent, efficacy in preventing progression of the disease, short and long-term adverse effects, costs, and patient burden:

*--Hydroxyurea (HU)* is an orally administered drug that can ameliorate some of the complications of the disease in both adults [4] and children [5], and increase life expectancy [6]. It is relatively well tolerated but requires routine monitoring, has to be continued indefinitely and may not offer protection against some of the long-term complications of the disease.

*--Bone marrow transplantation (BMT)* is the only therapy for SCD with curative intent, and involves replacement of the diseased bone marrow with bone marrow from a healthy matched donor. BMT cures a high proportion of patients transplanted from matched sibling donors, but is associated with risk of substantial short and long-term morbidity, long-term sequelae, and risk of mortality [7–9]. BMT is also only available to a small subset of patients with SCD who have an available matched donor.

*--Chronic blood transfusion (CBT)* involves regularly scheduled blood transfusions to decrease circulating sickle cells and is a proven therapy for both primary and secondary stroke prevention in SCD [10–12]. CBT is, however, associated with the potential for complications including transfusional hemosiderosis, alloimmunization, and the possibility of transfusion-associated infections.

Given the different benefits and risks of these treatments, the decision-making process regarding disease-modifying therapies in SCD is complex for both patients and families and healthcare providers. Interactions and discussions with healthcare providers are the primary modes by which patients and families examine and select between therapeutic options for SCD. There is however, a paucity of data, and hence a lack of understanding, on how physicians approach discussions regarding disease-modifying therapies in SCD. The approach or the philosophy of the physician to decision making regarding these treatment options is crucial in understanding the nature, quality, and consequences of decision making in SCD.

The premise of Shared Decision Making (SDM) [13] is that clinicians and patients make healthcare decisions collaboratively, using the best available information, closely examining the pros and cons of available treatment options, and carefully considering the patient’s personal values and preferences. SDM improves patient understanding of the expectations and consequences of decisions, and facilitates the achievement of outcomes consistent with patient preferences [14]. These features, some argue, render shared decision making an ethical imperative [15]. Proper execution of SDM, however, requires a culture that fosters patient engagement, an open dialogue between patient and physician, and guidance on how to weigh various influential factors [13]. Without sufficient research on how physicians approach these conversations, and on which factors are most influential on their decisions, it is difficult to effectively design systems that facilitate SDM. The goal of this study is, therefore, to gain insight into physician perspectives on decision making regarding disease-modifying therapies in SCD and to identify factors that may influence the approach that physicians take when it comes to decisions about disease modifying therapies.

## 2. Methods

### 2.1. Data collection

The data for this study were collected as part of a larger research effort to develop a web-based decision aid to help patients and families with SCD to make informed decisions regarding disease-modifying therapies. These interviews were conducted as part of decisional needs assessment interviews with patients, stakeholders, and physicians. This manuscript provides a theory-informed analysis of the interviews with physicians who care for patients with SCD.

The primary goal of the physician-focused interview script was to elicit physician perspectives on what patients need to make high-quality decisions regarding therapies for SCD; this approach was informed by the decisional needs assessment of the Ottawa Decision Support Framework[16]. Specifically, the interviewer sought to obtain physicians’ perspectives on patients’ (and/or family) knowledge, expectations, values and preferences in making these decisions. An additional goal was to discuss individual physicians’ philosophy and approach to therapies in SCD. Interviews were open-ended and semi-structured (Interview guide in S1 Appendix), and all interviews were recorded and transcribed verbatim. In these interviews, patient or parent, family or other caregivers were regarded as a single unit, knowing that with pediatric patients, the parent or caregiver was the surrogate decision maker.

Qualitative researchers recommend a sample size for a semi-structured interview of between 30–60 participants [17]. Given the breadth of topics explored in the interviews and the relatively limited number of experts in SCD and BMT, we sought to enroll as many physicians with expertise in SCD and BMT as possible. We therefore, enrolled a geographically diverse and nationally representative sample of physicians within the data collection timeframe of November 2013 to April 2014. Physicians were experts in the field of SCD and were recruited at national meetings or through national collaborative groups. Physicians were adult or pediatric hematology-oncology and/or bone marrow transplant specialists, and the majority were affiliated with academic medical centers. Of the 40 physicians enrolled, we were unable to schedule an interview with only one physician within the time frame of the study. The majority of these interviews were conducted by the lead author, but a minority, were conducted by other members of the research team in situations where the lead author was unavailable. In two cases, the physician interviews were incomplete due to technical difficulties with audio recording. In one case, the audio recording was missing, and it was not possible to obtain a transcript for analysis. The final sample of 36 interview transcripts served as the analysis sample.

Informed consent was obtained before all procedures, and all study procedures were approved by the Institutional Review Board (IRB).

### 2.2. Data analysis

In reviewing the interview transcripts, we used a thematic analysis approach [18], which provided a strategy for organizing and interpreting the data to “create a narrative understanding” of the physicians’ subjective experiences with medical decision making with SCD [18]. The first step was to read through the interview transcripts and to jot down memos in the margins. We used open coding techniques as outlined by Corbin and Strauss [19]. The focus of the coding process was to explore how physicians describe their approach to conversations with their patients and caregivers. More specifically, we investigated whether physicians enter discussions of treatment options as supporting patients and/or families in making decisions or, in contrast, to steer patients/families toward the treatment choices that they–the physician–viewed as best for the patient.

A coding scheme was developed using NVivo 11 software. Each code was defined and sustained throughout the analysis; these codes eventually developed into categories. From the coding process, we retained the categories that we believed held the most explanatory power. The primary categories, ‘Patient Characteristics,’ ‘Physician’s Perceptions of Patient,’ and ‘Decision Making Characteristics,’ were further analyzed for the degree of variation in the physicians’ attitudes towards physician-patient interactions. Additionally, transcripts were analyzed for factors that could influence decision making regarding disease-modifying therapies, including physician beliefs about patient expectations and patient perceptions of risks and benefits.

In the initial steps of analysis, we gave all data equal consideration [20]. We focused on variation across the data rather than frequency counts of concepts. Analysis concluded when we observed the replication of concepts[20]. Ultimately, we developed categories from consistent patterns in the data. Thematic saturation occurs when repeated refinements do not produce any new concepts relating to the research questions. Thus, thematic saturation was achieved when the team believed the development of categories addressed the research question[19].

Inter-coder agreement was achieved in three steps. One member performed the initial coding of the transcripts. As concepts developed, they were discussed and deliberated by the entire team. Once completed, a second team member coded all transcripts verifying the original coding scheme. Although laborious, repeating the coding process allowed the two team members to be completely immersed in the data, thereby enhancing the final analysis. After all these steps, disagreement occurred in a few transcripts, in which event a third team member served as arbitrator by also coding the transcripts in question. Results were discussed until the entire team came to a consensus.

The lead coder was a Ph.D. trained sociologist with experience in qualitative research and the second coder was a physician with an MS in research and with prior qualitative research experience, who verified the findings and framework developed by the lead coder.

## 3. Results

### 3.1. Demographic data

An approximately equal number of male and female physicians were interviewed. We classified physicians based on their primary expertise (SCD, BMT or both), and the age of patients they saw (whether primarily adult or pediatric). Demographic data of our study sample are reported in Table 1.

**Table 1:** Demographic Data.

Participant Characteristics (n = 36)	
Female sex, n (%)	17 (47.2%)
Primary Expertise, n (%)	
SCD	27 (75%)
BMT/Both SCD and BMT	9 (25%)
Age of patient primarily seen in practice, n (%)	
Pediatric	23 (63.8%)
Adult	11 (30.5%)
Not Reported	2 (5.5%)

### 3.2. The continuum of physician approaches to decision making

**Fig 1:**
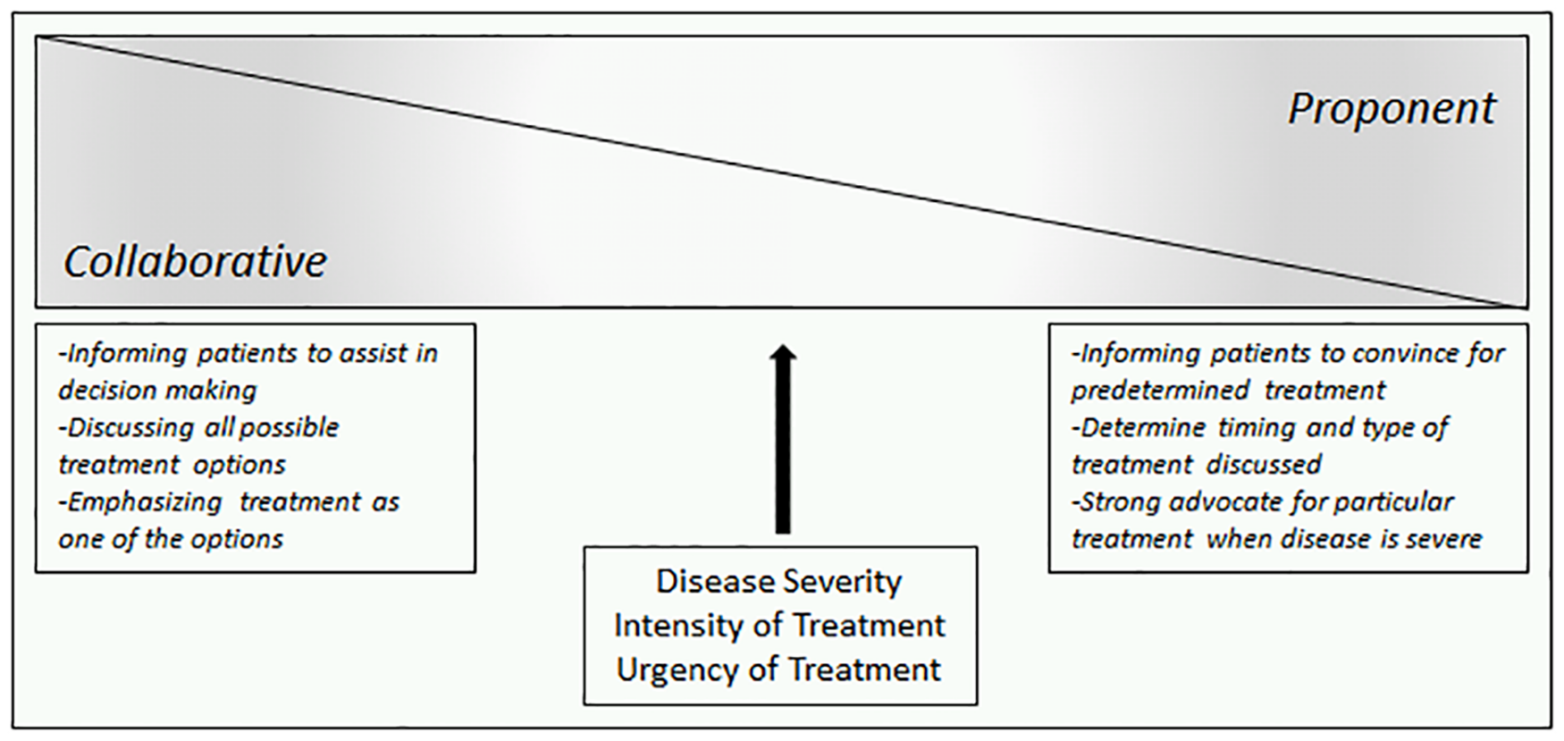
Continuum between collaborative and proponent approaches.

We identified a continuum of physician approaches for discussing disease modifying therapies for SCD. At one end of the continuum, we identified a collaborative approach (CA), in which the physician worked with the patient as an equal decision-making partner. At the opposite extreme, we identified a proponent approach (PA), in which the physician advocated for the treatment option that they believed to be appropriate. While the CA tended to discuss more than one treatment option, the PA focused on the treatment option believed to be appropriate by the physician. Both approaches, however, were characterized by concern for patient well-being and with a goal of achieving the best outcomes. As opposed to ‘belonging’ to a particular approach, which would suggest an ‘absolute’, physicians adopted approaches along the continuum between PA and CA (Fig 1).

In the CA, the physician emphasized the importance of ensuring that the patient and families were well-informed about risks, benefits and all possible treatment options so that a medical decision for treatment could be made. Physicians taking the CA approach often explicitly referred to how patients and caregivers require information about SCD and treatment options so that they can make a sound decision regarding treatment:

*“So I usually have the family [in for] two or three sessions of discussions with some introductory period discussing it*, *going through risks/benefits*, *probably a 20 to 30-minute discussion*, *and then some handout materials to read*, *and tell them then*, *‘We’ll have you come back again after you’ve had a chance to talk it over*, *think it over*, *read things*, *give them a website or two to look at as well and then sort of have a cooling off period a little bit*. *And at a second session*, *have us go over the things again and say*, *‘Are you still interested*?*’ Sometimes they are at that point*, *and sometimes they have decided not to [continue]*, *and then once in a while we go on for a third session.”*

(Pediatric Hematologist)

Another feature of the CA narrative was the tendency to describe the decision for treatment as ultimately belonging to the patient and family. In other words, the physician may have presented the treatment believed to be the best option, but would emphasize the patient’s/family’s agency in choosing a treatment plan that the patient himself believed was the best option:

*“It’s a really difficult thing*, *you know*, *and again that’s why I think this work is so important because it’s such a challenge to be able to honestly and openly help people to make a decision that there is no one right answer*, *you know.”*

(Pediatric hematologist)

In the CA, physicians frequently alluded to the importance of discussing all available treatment options rather than limiting information based on their perceptions of the patient or disease severity:

*“I think that you have the moral responsibility of introducing them to this [transplant]*. *I would write this on the website that this is an option*, *just potentially curative*, *and that families are encouraged to visit a stem cell transplant center and then look at their options and see the risks and the benefits for that.”*

(Pediatric Hematologist)

In contrast to the CA, at the other end of the continuum was the PA. PA narratives were characterized by an emphasis on getting the patient to adopt a treatment plan that was determined by the physician. While physicians using this approach still recognized the agency of the patient and/or family and recognized that the patient’s and/or family’s acceptance of and commitment to the treatment plan was necessary for long-term success, PA narratives suggested that patient values and preferences were considered as barriers to be overcome rather than valuable parts of the discussion:

*“You can impair function of the sperm [with Hydroxyurea]*, *but I don't really know if that's gonna translate to fertility issues or not*. *Much of that can be reversible*, *so I don't emphasize it*, *but I will mention and then I think the reaction that there are some questions about impact on fertility*. *But*, *I don't really go into that very much*, *because I don't [want to] warn people off Hydroxyurea.”*

(Pediatric Hematologist)

Frequently, physicians reported that recurrent meetings with patients and/or families were necessary to fully explain therapeutic options. Whereas the CA held multiple discussions with the goal of patient education, the PA focused on using each meeting for persuading the patient and family to accept the recommended treatment plan:

*“Now*, *I had one parent that I kept dragging back into my office until she finally put her son on Hydroxyurea for pain.”*

(Pediatric Hematologist)

With this approach, if the physician was not an advocate of a treatment option, that option was likely not to be discussed with the patient. This was seen especially frequently with BMT, which was often not discussed with the caregiver family unless the patient met certain severity criteria:

“*So I would only recommend it [BMT] to patients who are at very high risk of death*, *or their quality of life is severely impaired.”*

(Adult Hematologist)

As evidenced by our interview data, the PA is characterized not only by advocating a specific treatment plan but also by a reluctance to discuss particular treatment options when the physician believed the patient was not a good candidate from either a clinical or compliance perspective.

As we have noted, physicians adopted approaches along the continuum between PA and CA. This led us to our second research question: what were the factors that influenced physician decision making and, in particular, the type of approach adopted in interacting with patients and families/caregivers?

### 3.3. Characteristics influencing physician approach

**Fig 2:**
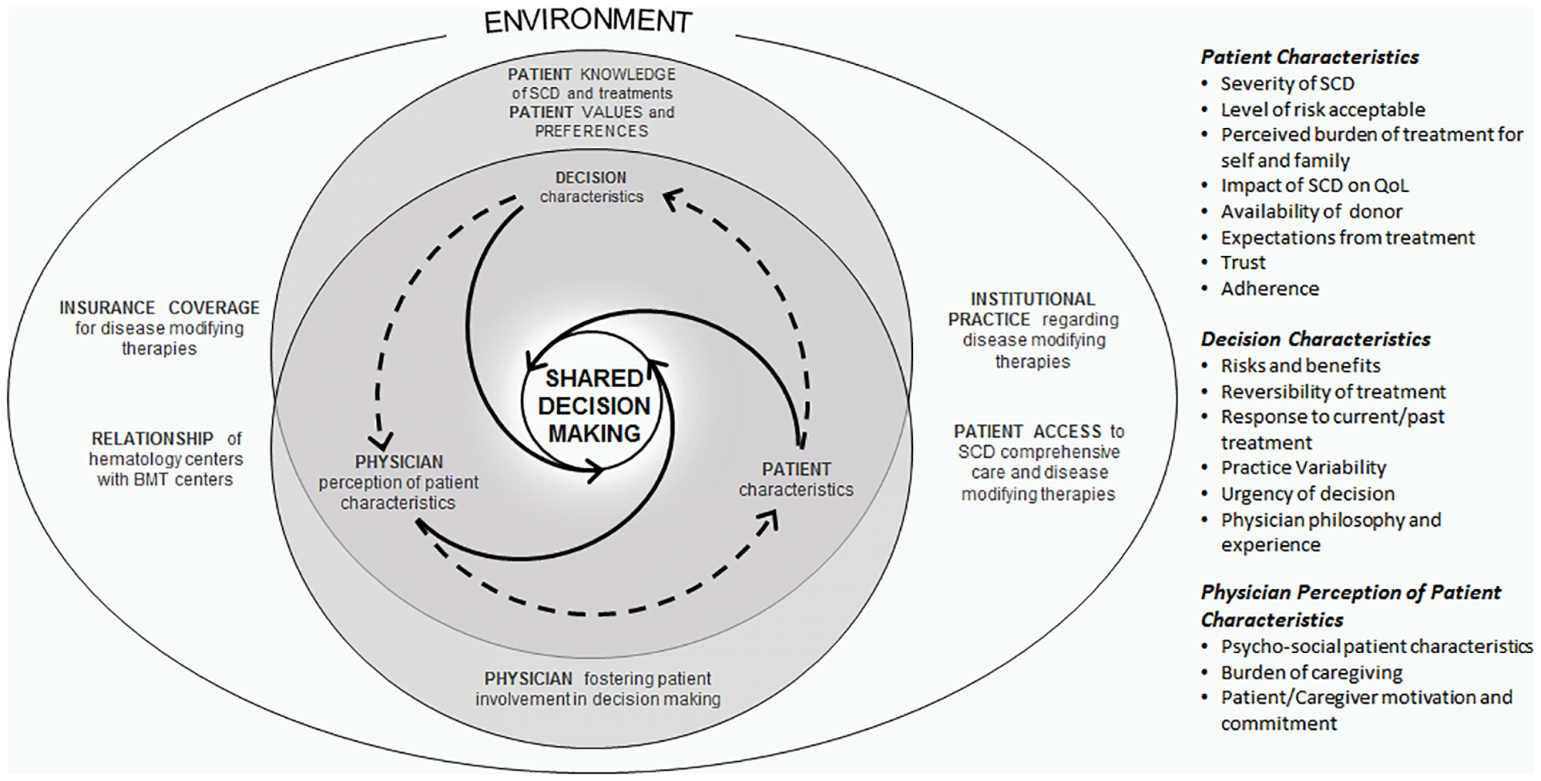
Model depicting factors influencing decision making regarding disease-modifying therapies in SCD.

Our interview data revealed four primary categories of factors that impacted the physicians’ approach to interacting with the patient and/or family in decision making regarding disease-modifying therapies for SCD: patient characteristics, physician perception of patient characteristics, decision characteristics and organizational factors (Fig 2). The interplay between these categories guided the physicians’ narrative approach. We structure our discussion around each of the three treatment options. For each, we present how physicians describe their decision-making process, identify influential factors (across all four categories), and discuss how these combinations affect choice of approach.

While we identified several different factors that influenced a physician’s approach, one of the most important was the severity of SCD for the individual patient. Physicians tended to present Hydroxyurea as a viable treatment option when the patient presented increased clinical symptomatology and disease severity. While there was variability in whom physicians believed HU should be prescribed to and whether they primarily adopted the CA or the PA, we found that the PA was more common with increased disease severity. Physicians indicated that they emphasized the benefits of HU, including a decrease in acute complications such as pain episodes and acute chest syndrome, a decrease in hospitalizations, improvement in hemoglobin, and improvement in fatigue and quality of life; they also made sure to communicate that these benefits would slowly occur over time. Physicians cited research most frequently as justification for treatment, but also to educate the patient.

Many physicians said that patients were apprehensive to select HU treatment because it is a chemotherapeutic medication, and patients had concerns about developing cancer with long-term use. Some physicians also reported that patients were concerned about the potential impact of HU on fertility, particularly the effect on sperm counts. While physicians discussed with patients’ other side effects, such as bone marrow suppression, they were typically presented as being mild and reversible after discontinuation of the medication. Physicians identified the ‘inconvenience’ of taking the medication, i.e., having to come for frequent follow-up appointments, lab draws, missing school or work for additional clinic appointments or the burden of taking additional medications as barriers to the use of HU.

As disease severity increased, physicians were more likely to discuss the possibility of BMT with patients/families, although we found that presentation of BMT was not uniform among providers. A key factor influencing presentation of BMT was the availability of a potential donor. However, there was significant variability amongst physicians in how these two factors (disease severity and donor availability) impacted discussions of BMT with patients and/or families. While many physicians agreed that a candidate with severe disease who had a matched sibling should be offered BMT, there was less consensus on the use of BMT for the asymptomatic or mildly symptomatic child with a matched sibling. Some, but not all, physicians mentioned response (or lack thereof) to current therapy as an important consideration in their decision to discuss transplant. Additionally, physicians also considered the perceived burden of SCD on a patient and family in determining whether to discuss BMT.

With BMT, we found that a physician’s attitude toward the use of the treatment, and their prior experience with BMT were important factors in the decision making process. Poor outcomes of previous patients, such as severe morbidity due to graft vs. host disease or death, seemed to exert a powerful influence on risk appraisal and physicians’ later decisions. We also found that if a physician felt that HU was the best treatment option and was following the PA, he or she tended to not mention BMT until the patient’s disease severity reached a threshold that they thought justified the risk of BMT.

Having realistic expectations regarding the transplant process was another aspect discussed by physicians, especially the knowledge that the benefits from a successful transplant would occur over time. Lastly, physician perception of other factors, such as patient commitment, ability to adhere to a medical plan, psychosocial factors, and financial factors was part of the decision making process, although these had different implications for different physicians:

*“My belief has been that I will not penalize the child for something that the parent may be responsible for*, *so I’ve always felt that that alone should not be a reason for denying a child a transplant*. *I think as a medical system*, *we should be able to work on those issues.”*

(Pediatric BMT physician)

When presenting BMT as a treatment option, several physicians commented on the importance of giving patients and/or families sufficient information and time to make an informed decision, which, several noted, often required multiple meetings. Physicians wanted to ensure that patients and/or families had an adequate understanding of risks of BMT, such as death, graft vs. host disease, potential infertility, long-term adverse effects of chemotherapy such as secondary malignancies, and the potential for the graft to fail. Physicians wanted patients to understand that BMT is a serious procedure with the potential for morbidity and mortality, and that it involves a major lifestyle change, time off from school or work, or even potential relocation to another city.

One physician commented,

*“Our goal is*, *at the end of not just one*, *but several conversations*, *is for them to have a better understanding of the pros and cons of transplant*, *for them to go through a period of perhaps doubt and then discuss with family members that they wish to and then come to terms with the actual process*, *… so if we see none of that*, *it makes us a little concerned that perhaps we haven’t done as good of a job as describing the process to them*, *so we do it over several sittings*, *so to speak.”*

(Pediatric BMT physician)

*“I think they need time to make sure this is the right decision to do*.”

(Pediatric BMT physician)

While physicians reported that patient expectations of BMT varied, they reported that the patient and/or family’s underlying goal was to obtain relief from their suffering, especially vaso-occlusive pain, and to be cured of their disease, resulting in a better quality of life. Physicians felt that patients and/or family’s perception of the risks of BMT, were variable and dependent on individual values. For example, while some patients accepted the risk of infertility, many would not proceed with transplant once they learned it was an adverse effect.

Many physicians reported especially great difficulty in effectively conveying the extremity and severity of the risk of GVHD to patients, which, some reported, patients tended to view as little more than a potentially uncomfortable side effect of the procedure:

*“I don’t think they completely understand but when you describe you know graft vs*. *host disease to them you know*, *some see it as a consequence*. *I don’t think they fully understand how debilitating it could be and a threat to their life…*.*I don’t think they understand morbidity involved with GVHD”*

(Adult BMT Physician)

Physicians described how the decision making timeline for BMT for patient and/or families could range from months to years, although some physicians did note that patients who had personally witnessed death and severe complications from SCD were more likely to proceed with BMT sooner. Physicians also reported that patients speaking with other patients and/or families about their disease characteristics and their experiences with various treatment options were particularly powerful in decision making, for both HU and BMT, but particularly for BMT.

In contrast to discussions about BMT, which were very likely to follow CA, when physicians recommended chronic blood transfusions, they almost always reported interactions with patients/families that were consistent with PA:

*“[I] sell transfusions a little bit*, *more as this is a standard of care… I think I come about it a little more predetermined in what I want*, *as opposed to having a discussion about [transfusion].”*

(Pediatric Hematologist)

All physicians utilized CBT for primary or secondary prevention of stroke. When patients presented with less standard indications such as recurrent acute chest syndrome, chronic pain, priapism, or leg ulcers but cited non-response or failure of standard therapies, some but not all physicians recommended CBT. Physician perspectives highlighted the commitment that CBT entailed as a treatment, and the burden it placed on patients and families.

Most physicians highlighted the challenges in helping patients and/or families understand the complications associated with SCD as well as the challenges of chelation therapy and adherence:

*“Patients hear about it*..*but they really don’t*, *they’re not impressed by it…they’re not impressed when we tell them all the consequences of iron overload*, *‘Yeah*, *yeah*, *I know all about that.’”*

(Pediatric Hematologist)

### 3.4. Influential factors beyond patient and decision characteristics

Many physicians indicated that decision making was facilitated if patients and/or families had a strong relationship with their physician, trust in the medical system, and a perception that their physician would help them overcome barriers in selecting and obtaining a given treatment option. Our interviews identified many such barriers to the decision-making process. In particular, patients/families often did not fully understand the ongoing end-organ damage or early mortality arising from untreated SCD, especially if they had no outward or recurrent acute complications of the disease. Physicians felt that improved understanding would lead patients to more readily adopt disease modifying or curative therapies.

Some physicians also cited a lack of trust or active distrust in the medical system as a barrier, particularly when a therapy was part of a research or clinical trial. Organizational factors, such as ease of access to an SCD comprehensive center or the lack of a working relationship between hematologists and a BMT center, in some cases also appeared to present barriers to the adoption of these therapies. Another potential barrier was physician philosophy regarding the proposed treatment plan; this appeared to play a role in both HU and BMT. Finally, perception of the therapeutic option in the SCD patient community also influenced a patient’s decision to adopt that treatment.

### 3.5. Framework for decision making regarding disease-modifying therapies in SCD

Based on the data and using Joseph-Williams’ model of patient-reported influences on individual capacity to participate in shared decision making [21] as an inspiration, we propose a model to illustrate the influence and interplay of patient characteristics, decision characteristics, physician perspective, and organizational (or environmental) factors, as discussed above, on decision making (Fig 2). In the outermost ring, we identify organizational factors; these are overarching determinants that affect both the physician and the patient.

The next layer illustrates the interplay between decision characteristics (often treatment dependent), patient characteristics, and physician-patient perception of patient characteristics; it also references Elwyn *et*. *al*.*’s* proposition [13] that all three of these factors are relevant in developing both patient knowledge of SCD and an environment for patient engagement.

The innermost ring of our model represents the ideal outcome of SDM. This model shows how physicians, patients, and the treatment decision each contribute to SDM and also influence each other.

## 4. Discussion

Our research describes how physicians with expertise in sickle cell disease may take different approaches when it comes to involving patients in decision making regarding disease-modifying therapies. In some circumstances, physicians take a PA, in which they advocate for a given treatment plan and seek to obtain patients’ consent. In others, physicians take a CA, in which they actively seek to engage patients and/or families in the decision-making process and work together to reach a decision in line with the patients’ values and preferences. We also identify factors that influence physician decisions and approaches, including a variety of patient, treatment, and disease severity characteristics as well as institutional frameworks.

While previous research has focused on patient perspective of treatment-related decision making, there is a paucity of research investigating the physician perspective of treatment-related decision making. This is the first study, to our knowledge, which explores the physician perspective of how they approach discussions regarding disease-modifying therapies for SCD. Increased knowledge of the factors that influence physicians’ behavior and decisions will allow more effective research on how to promote and facilitate SDM.

Additionally, there is not yet an established framework for SDM for SCD in particular, and we believe that decision making in SCD, while drawing from a framework similar to other chronic illnesses, may have some unique characteristics. SCD is an inherited blood disorder beginning in infancy, and there is often not a single decision point in SCD. Rather, as described by Lipstein *et*. *al*. [22], the decisions are part of a longitudinal process without a clear beginning and end, where each decision leads to the next. In addition, the consequences of any decision, or lack thereof, is associated with downstream consequences for the clinical course of the disease. SCD is especially complicated by increases in disease-related complications with age, as well as by its consequences for decreased lifespan. As mentioned earlier, SDM may be further complicated by the fact that SCD disproportionately affects racial and ethnic minorities and those with low socioeconomic status [23]. Although not explored in our study, race may potentially impact SDM through its influence on the patient-provider relationship as well as because of the distrust of the medical establishment that is prevalent amongst African-Americans[24]. Research has shown that patients with SCD have lower trust in the healthcare system and perceive discrimination from healthcare providers [25], which is associated with poorer patient-provider communication[26]. Lower socioeconomic status is also associated with lower levels of health literacy [27], which inhibits full patient participation in decision making. Lack of patient trust, perceived discrimination, and lower socioeconomic status associated with lower health literacy are all factors that can potentially act as barriers to SDM in SCD. Patient-related barriers have also been found to influence adoption of therapies like HU in SCD. Lastly, since the disease starts in infancy, and continues through the lifespan of the individual, there are developmental considerations over the lifespan that need to be taken into account in the decision making process.

Our interviews highlight that physicians used different approaches in decision making depending on the proposed treatment, and that these approaches lie on a continuum, as other authors have also described [28]. We believe that this variation in approach to HU and BMT is a function of the nature of the therapies themselves. These treatments have different goals: HU is a disease-modifying, non-curative treatment, whereas BMT is a treatment with curative intent. We believe that the increased evidence base for HU along with its ease of administration, wider availability, and safer risk profile compared to BMT may sway physicians towards HU rather than BMT. Our data suggests that physicians may fail to present patients with all available treatment options due to their personal philosophy about a particular treatment option. Wennberg *et*. *al*. have suggested that attitudes of individual physicians, or “practice style factor” [29], as well as evidence base or population characteristics, contribute to practice variation. This appeared to be especially relevant in discussions regarding BMT; we found that physicians’ prior experience or outcomes with the treatment was a factor in influencing discussions regarding BMT with their patients. While the decision on whether to pursue chronic blood transfusion was explored in less detail, we found that physicians typically presented it using the PA. Even though physicians presented the risks and benefits of CBT, once they determined that a patient needed CBT they did not believe that there was the possibility of an alternative decision. We believe this is because CBT is considered primarily in clinical scenarios where not adopting the therapy is associated with a high risk of severe morbidity and potential disability.

We integrate these results into a framework that depicts the interplay of physician, patient, and treatment characteristics on SDM in the context of external and institutional influencing factors. This framework is supported in the literature, where level of support for SDM varies by clinical scenario, treatment decision, and patient characteristics [30]. Research also suggests that with the availability of evidence-based guidelines in favor of a particular treatment option in a given scenario, physicians may feel pressured to follow such recommendations and thus may be less likely to involve patients in decision making [31]. We see this most strongly with the approach to CBT and to a lesser extent with HU. Additionally, our findings corroborate research indicating that situational factors, such as clinical scenarios and the urgency of decision making, influence SDM [32]. Finally, studies have shown that physician attitudes and beliefs determine translation of guidelines into clinical practice [33]; we found similar results in our study regarding HU and BMT.

The primary strength of this study is that it provides a detailed analysis of the physician perspective on decision making in SCD, an area that is not often addressed in the literature surrounding SCD or SDM. The study also provides insights into factors that may influence this process, such as severity of disease, type of clinical decision as well as urgency of treatment. Focusing on how physicians navigate these complex decisions is critical, since they play a large role in determining patient treatment plans. Our qualitative study design allowed for a rich description of these complexities, and enabled us to create a framework of how physicians make decisions in SCD. The interviews highlight the challenges of decision making in the real world, where factors beyond scientific evidence and clear consideration of individual preferences determine therapeutic options. Another strength of this study was its relatively large sample size, compared to most qualitative studies, and the depth in which approach to treatment options was discussed by physicians in this study. The physicians were from different regions of the U.S., and our resulting ability to account for practice variations between different regions of the country further contribute to strengthened external validity.

Our findings are limited by the high degree of specialization of participants; many of the physicians interviewed are regional or national experts in sickle cell disease and/or bone marrow transplant, and most are associated with academic centers. Therefore, their opinions may not fully represent the views of all physicians or hematologists who treat patients with SCD. Furthermore, these narratives focus on the physician perspective rather than measurable use of SDM; while they provide a unique perspective into how physicians approach decision making, they were not meant to objectively evaluate how frequently physicians engaged in SDM under different scenarios. Lastly, as with any qualitative study, there may be a social desirability bias that caused physicians to report narratives that deviate from their actual practices.

## 5. Conclusion

In conclusion, we demonstrate that physician perspectives on medical decision making regarding disease-modifying therapies in SCD vary from Collaborative to Proponent Approaches, and that approach is influenced by a variety of patient, disease severity, and decision characteristics as well as institutional frameworks. These insights fill a gap in the literature on physician perspectives on SDM in SCD, which may differ from SDM in other medical contexts. Our results highlight the influence of physicians in decision making and provide rationale for future studies focusing on physician-patient encounters regarding disease-modifying therapies in SCD. Continued research on the role of physicians in SDM will facilitate the design of systems that foster patient engagement and encourage open dialogues, benefitting both patients and caregiver families.

## Supporting information

S1 Appendix Interview guide. (DOCX)
