# Supplementary material for: Proponent or collaborative: Physician perspectives and approaches to disease modifying therapies in sickle cell disease
Source: PLoS One. 2017 Jul 20;12(7):e0178413. doi: 10.1371/journal.pone.0178413 (PMC5518995; doi:10.1371/journal.pone.0178413)
Supplement: S1 Appendix — (DOCX) [file pone.0178413.s001.docx]

I would like to discuss the decision making process for patients and caregivers with [treatment option] as a treatment for sickle cell disease.

1. What is your philosophy regarding [treatment option] for patients with sickle cell disease?

*Suggested Probes*: disease characteristics, disease severity, patient selection, genotypes considered, factors considered in decision making, perception/discussion of risks and benefits, adherence

If discussing BMT- additional factors in decision making (disease severity, genotypes, donor type, HU use, adherence), perception of risks (such as death GVHD, infertility) and benefits

1. Can you describe the decision making process [treatment option] for patients and their caregivers.

*Suggested Probes:* Educational process, who is involved, what is considered?

1. What do patients perceive as benefits and risks for [treatment option]. What are their expectations from [treatment option]?

*Suggested Probes:* understanding of risks/benefits, type of expectations and if realistic

1. What factors affect decision making regarding [treatment option].

*Suggested Probes:* patient values, perception of risks and benefits, facilitators, barriers

1. Do patients have any regrets after choosing [treatment option] (if applicable)

Probes:

1. What should be included in a web-based decision tool? And how should this be presented?

*Suggested Probes:* Text, testimonials, presentation of research, presentation of probabilities, any [treatment option] specific considerations, etc.

1. Is there anything else you would like to mention?

Thank you for your time and participation!
